# On the extension of the use of a standard operating procedure for nicotine, glycerol and propylene glycol analysis in e-liquids using mass spectrometry

**DOI:** 10.18332/tid/191823

**Published:** 2024-09-11

**Authors:** Alessia Turina, Alice Passoni, Silvano Gallus, Alessandra Lugo, Walther Klerx, Reinskje Talhout, Ranti Fayokun, Constantine Vardavas, Enrico Davoli

**Affiliations:** 1 Laboratory of Mass Spectrometry, Istituto di Ricerche Farmacologiche Mario Negri (IRCCS), Milan, Italy; 2 Department of Medical Epidemiology, Istituto di Ricerche Farmacologiche Mario Negri (IRCCS), Milan, Italy; 3 Department for Chemical Analysis, Tobacco & Drugs, Center for Health Protection, National Institute for Public Health and the Environment (RIVM), Bilthoven, The Netherlands; 4 No Tobacco Unit, Health Promotion Department, World Health Organization, Geneva, Switzerland; 5 Hellenic Cancer Society, Athens, Greece

**Keywords:** nicotine, mass spectrometry, standard operating procedure, e-liquids

## Abstract

**INTRODUCTION:**

Standard operating procedures (SOP), accessible to several laboratories, are essential for product verification. EU-JATC (European-Joint Action on Tobacco Control) SOP and the WHO TobLabNet (World Health Organization Tobacco Laboratories Network) SOP (SOP11) are available standard methodologies to measure nicotine, glycerol, and propylene glycol, and propose mass spectrometer (MS) as an alternative method to flame ionization detector (FID). This study conducted a comparison between FID and MS concentration results, following the MS method described in SOP11.

**METHODS:**

In May 2020, five test e-liquids in replicates (A-E) were prepared at the Istituto di Ricerche Farmacologiche Mario Negri and sent, with SOP 11, validation document and results datasheet to 32 different laboratories all over the world from WHO TobLabNet and EU-JATC (18 from JATC, ten from WHO TobLabNet and four academic laboratories). Among thirty-two independent laboratories that participated in the study, results were received from 30 laboratories.

**RESULTS:**

The e-liquids analyses, using the two approaches, were compared. Of the 30 laboratories surveyed, 21 utilized the FID approach exclusively, 7 opted for MS detection, and 2 employed both methods. The findings demonstrated that the gas chromatography-mass spectrometry (GC-MS) method offers comparable analytical capabilities regarding accuracy and precision for nicotine, glycerol, and propylene glycol to the FID approach. Through Pearson’s correlation test with r≃1 showing a positive correlation between GC-FID and GC-MS data, and the Student’s t-test, no significant differences between the two approaches were revealed, showing p>0.005 for almost all three analytes in all samples.

**CONCLUSIONS:**

This study indicates that it is possible to apply the available EU-JATC SOP and the WHO TobLabNet SOP11 even in laboratories that do not have access to an FID, for example, to analyze flavors, trace compounds or carcinogenic, mutagenic, or toxic for reproduction (CMR) in electronic cigarette liquids.

## INTRODUCTION

Over the last decade, electronic cigarettes (e-cigarettes) have become extremely popular among both adults and youth^1^. Among young people, e-cigarette use is very common. It is noticed that, in Europe, the highest prevalence of e-cigarette users was among those aged 10–24 years, followed by 25–39 years, 40–65 years, and those >65 years^2^. Recently, an increase in e-cigarette use has been observed, and one of the main causes is related to the public perception that vaping is harmless, or at least less harmful than cigarette smoking^3,4^. E-cigarettes are characterized by a liquid solution that typically contains propylene glycol (PG) and glycerol to generate vapor and act as a carrier for nicotine and flavorings^5^, which reduces the perception of bitterness and harshness, and increases the willingness of non-users to try e-cigarettes^6-8^.

It is crucial to emphasize that vaping products carry risks, given that inhaling vaping aerosol exposes individuals to potentially harmful chemicals^9,10^. Vaping has been linked to lung injuries that manifest with various symptoms, impacting not only the respiratory system but also potentially affecting the cardiovascular, gastrointestinal, and systemic systems^11^. The dangers of e-cigarette vaping include exposure to toxic substances such as heavy metals, volatile organic compounds, and ultrafine particles^12,13^. Some studies showed that fine and ultrafine particles present in e-cigarette aerosols could cause health concerns for users and secondhand smokers^14^. E-cigarettes have been found to release elevated amounts of particle number concentration (PNC) and fine particulate matter (PM2.5), and these emissions are closely associated with the composition of the e-liquid, including factors such as the propylene glycol/vegetable glycerine (PG/VG) ratio and nicotine content^15^.

Nonetheless, despite these clear pieces of evidence, there has been an increase in the popularity of e-cigarettes, causing a significant public health issue. In Europe, The Tobacco Products Directive (TPD) (2014/40/EU) was introduced to reduce the burden of tobacco-related illnesses and deaths and to improve the functioning of the internal market for tobacco and related products^16^. In this context, the Joint Action on Tobacco Control 1 (JATC1), a European Commission-funded project, was designed to provide support in implementing the TPD across the EU Member States (EU-MS)^17^. Within the JATC, Work Package 8 (Laboratory Verification, Collaboration, and Analyses) was designed with the aim ‘To develop collaborations across EU-MS independent laboratories and identify suggested methodologies and test analyses for reporting homogenization’^18^.

Standard methods for analyzing components in e-cigarette liquids are available. ISO 20714, published in 2019, was a standard operating procedure (SOP) for determining nicotine, glycerol, and propylene glycol in e-liquids^19^. There is a pressing need for an independent standardized method unrelated to the tobacco industry to ensure accurate product analysis and verification. This necessity prompted the development of the EU-JATC SOP in 2020^20^. At the same time, the World Health Organization (WHO) also needed an independent method, and the WHO Tobacco Laboratories Network (TobLabNet) SOP11 was published in 2022^20^.

For the JATC and WHO methods, a network of independent laboratories was created to evaluate the method’s reproducibility and repeatability and the inter-laboratory variability. Most laboratories participated in the collaborative study to validate the method using gas chromatography coupled with a flame ionization detector (GC-FID)^18^. However, some laboratories also used mass spectrometry (MS) detection as an alternative method to the GC-FID method, as described in EU-JATC SOP and SOP11 procedures. This was important because laboratories conducting analyses of e-cigarette liquids often also analyze other trace components where mass spectrometry is crucial. Therefore, there is an increasingly common scenario where the analysis of nicotine, glycerol, and propylene glycol is conducted in laboratories without access to FID but equipped with MS. It is important that these laboratories can also conduct analyses following accepted standard procedures.

The current study aimed to compare the MS and FID approach and to assess the degree of agreement among independent laboratories in measuring the content of nicotine, glycerol, and propylene glycol in e-liquids using the method based on GC-MS. This research contributes to a comprehensive understanding of the analytical challenges posed by e-cigarettes and underscores the importance of reliable testing methods in ensuring the safety and regulation of these products.

## METHODS

### Study design, testing and training in e-liquids

Within the comparative cross-sectional study, five different test e-liquids were prepared in the Istituto di Ricerche Farmacologiche Mario Negri in Milan (Italy), using different nicotine, glycerol, and propylene glycol concentrations, within values that are generally found in commercial e-liquids (Table 1). Four liquids were prepared gravimetrically, and one was a pool of commercial e-liquids. All samples were prepared in large volumes, mixed overnight in a horizontal shaker to obtain a homogeneous solution, and stored at 5°C in dark conditions until the instrumental analysis.

**Table 1:** Nicotine, glycerol, and propylene glycol concentration in e-liquids samples sent to 32 laboratories worldwide from WHO TobLabNet and EU-JATC, in May 2020

*E-liquids (and replicates)*	*Nicotine (mg/mL)*	*Glycerol (mg/mL)*	*Propylene glycol (mg/mL)*	*Water (mg/mL)*
E-liquid A (A1-A2)	0.25	568.0	568.7	
E-liquid B (B1-B2)	5.08	213.9	855.0	
E-liquid C (C1-C2)	9.46	771.4	277.9	110.2
E-liquid D (D1-D2)	21.27	321.5	749.9	
E-liquid E (E1-E2)*	approx. 10	approx. 360	approx. 570	
Training e-liquid	10.02	816.7	233.4	116.7

* E-liquid E was a pool of commercial e-liquids and therefore an approximate concentration of analytes was derived from the declared composition on the label.

Test e-liquids A-E were sent to 32 different laboratories worldwide from WHO TobLabNet and EU-JATC (18 from JATC, ten from WHO TobLabNet, and four academic laboratories). Countries involved in this project were Austria, Germany, Belgium, The Netherlands, Spain, Greece, Ireland, Latvia, France, Luxembourg, Slovenia, Hungary, Italy, USA, Indonesia, Costa Rica, Burkina Faso, Japan, China, South Korea, Bulgaria, and Singapore.

E-liquid concentrations were blind to participants, and replicates of each concentration level were supplied to overcome problems due to possible losses during delivery or package damage. An extra training e-liquid was prepared for laboratories that requested it and sent in advance to train laboratories in practical issues regarding this method. These e-liquids were shipped using the SOP method, containing instructions on conducting the analysis and the SOP protocol, and describing how this study would be conducted and its timelines.

We received analysis results from 30 out of 32 laboratories initially involved. The results were presented in a designated Microsoft Excel template file, anonymized by the organizers, containing details about the equipment used (such as the type of GC detector, GC column, chemicals employed, FID or MS method, etc.).

### Standards and chemicals

The e-liquid samples were prepared using nicotine (CAS 54-11-5), purity >98%, glycerol (propane-1,2,3-triol, CAS 56-81-5), purity >98%, and propylene glycol (propane-1,2-diol, CAS 57-55-6), purity >98%. The solvent used to prepare standards was propane-2-ol (CAS 67-63-0).

Internal standards used were quinaldine (2-methylquinoline, CAS 91-63-4) or n-heptadecane (CAS 629-78-7) for nicotine and 1,3-butanediol (CAS 107-88-0) for glycerol and propylene glycol. All chemicals were from Sigma-Aldrich, Saint Louis, MO.

### Analysis by GC-MS

In SOP11 (Annex 2), the use of mass spectrometry is mentioned as an alternative measurement technique to FID, where an example of instrumental settings is reported. E-liquid samples for MS analysis are supposed to be prepared in accordance with sample preparation for FID analysis as outlined in the SOP 11^20^. The main points of sample preparation are reported as follows. E-liquids are brought to room temperature, homogenized using the vortex mixer for 30 s, and eventually sonicated to remove air bubbles before opening the container. A 100 μL of e-liquid is added to 9.9 mL of diluent solution made of propane-2-ol containing 0.5 g/L of nicotine internal standard, quinaldine (or heptadecane, mostly used for FID), and 2 g/L of glycerol and propylene glycol internal standard 1,3-butanediol. Due to the high viscosity of e-liquids, to add e-liquids A-E to the diluent solution, positive displacement pipets are used, or, when not available, e-liquids are weighed using density. Aliquots of samples A-E are then transferred into vials.

GC conditions are listed in the SOP11^20^. Briefly, 1 μL samples and standards are injected into the GC using one of the capillary columns listed in the procedure (DB-ALC1). Helium carrier gas is run with a flow of 1.5 mL/min through a split injector (50:1 split) at a temperature of 225°C. The oven temperature is increased by 40°C/min from 140°C (held for 5 minutes) to 250°C (held for 4 min). Supplementary file Table 1 details the nine laboratories that used the MS approach and the DB-ALC1 or equivalent columns used.

The MS source and transfer line are kept at ≥180°C. The MS analysis is run in the single-ion-monitoring mode at m/z (mass divided by charge number) values 61 (quantifier) and m/z 45 (qualifier) for propylene glycol; m/z 61 (quantifier) and m/z 43 (qualifier) for glycerol; m/z 161 (quantifier) and m/z 84 (qualifier) for nicotine; m/z 143 (quantifier) and m/z 115 (qualifier) for quinaldine; m/z 72 (quantifier) and m/z 75 (qualifier) for 1,3-butanediol.

Data analysis and calculations are based on the calibration curve approach, using the ratios of the peak areas of the quantitation ion for nicotine, glycerol, and propylene glycol and their respective internal standards and plotted against their concentrations.

### Statistical analysis

The inter-laboratory accuracy and precision were calculated. Accuracy was considered optimal if the value was below 15% and acceptable between 15% and 20%^21^. Accuracy results were obtained for all five different concentration samples. Precision value was obtained, and the coefficient of variation (CV%) was calculated. Results should fall within the range of ±15%. ISO 7525^22^ reported that the test of Cochran and that of Grubbs are recommended to identify outliers. These tests were employed at significance levels of α=0.05 and α=0.01 to detect outliers and stragglers, respectively. A value is considered a straggler when the test statistic lies between its 5% and 1% critical values. At the same time, this value is a statistical outlier when the test statistic exceeds its 1% critical value^23^.

Correlation and linear regression techniques were applied to quantify the association between FID and MS data. We calculated the correlation expressed as a correlation coefficient (r), quantifying the strength of the linear relationship between paired variables. Assuming that variables x and y are normally distributed, we used Pearson’s correlation test to verify the linearity, through the r-value, between the MS and FID methods. The coefficient (p) was also computed to verify a linear relationship between tested variables. Following the confirmation of normality and homogeneity of variance using the Shapiro test and the Levene test, respectively (except for samples A and E in the analysis of propylene glycol), the Student’s t-test was applied to confirm no significant difference between the two methods^24^. Prism Software was used to perform the two-tailed statistical tests described above.

## RESULTS

All sample analyses were conducted on the same day by the same operator, utilizing identical calibration parameters. Each sample underwent analysis in replication. Most of the results were obtained through the GC-FID method. Among the 30 laboratories, 9 conducted GC-MS analysis: two used GC-MS in addition to GC-FID, five exclusively used GC-MS, and two employed GC-MS only for nicotine analysis. Due to the absence of duplicates, results from the UA and UD laboratories (apart from sample B for propylene glycol analysis) were not considered.

### GC-MS results

**Figure 1:**
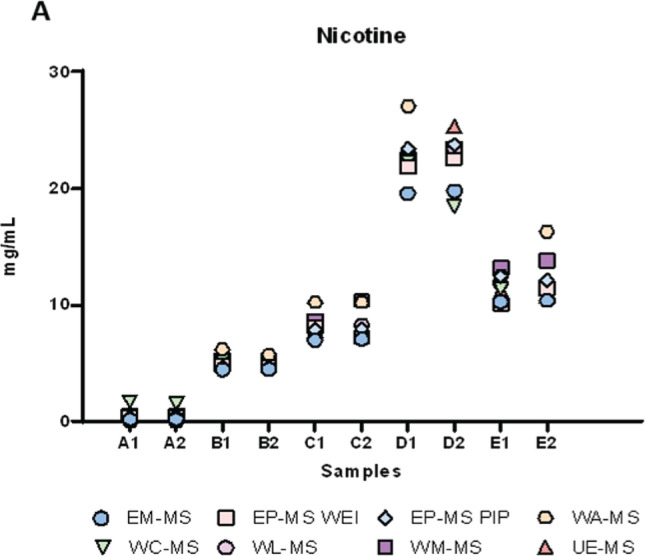
Distribution of participants’ concentration (mg/mL) results for nicotine

**Figure 2:**
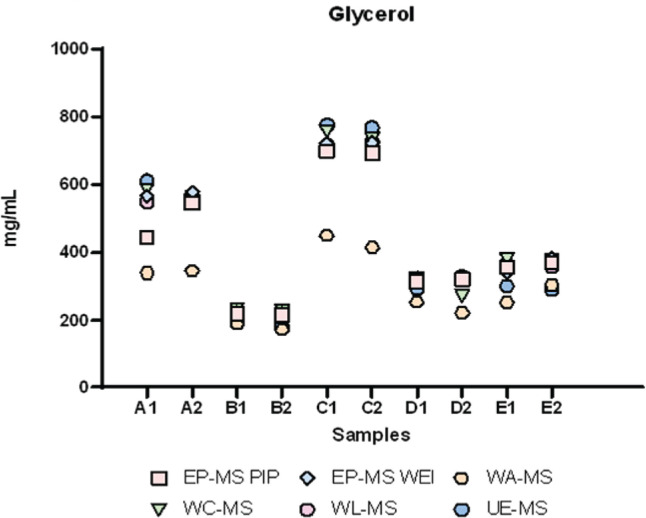
Distribution of participants’ concentration (mg/mL) results for glycerol

**Figure 3:**
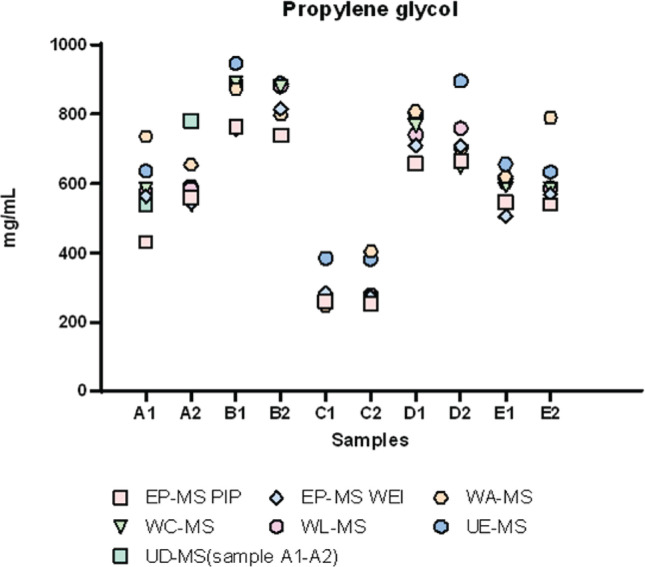
Distribution of participants’ concentration (mg/mL) results for glycol

GC-MS results are reported in Figures 1–3. Here, the distribution of participants’ results for nicotine (7 laboratories out of 9), glycerol (5 laboratories out of 9), and propylene glycol (6 laboratories out of 9) in samples A-E are presented. As these Figures show, the greatest challenges were encountered in determining glycerol and propylene glycol out of the three analytes. Several laboratories provided results for these two analytes that significantly differed from the average values.

*Statistical evaluation for GC-MS*

Accuracy results are reported in Table 2. Regarding nicotine, accuracy results from all laboratories for sample A exceeded ±20% from the declared values. In contrast, for the other samples (B, C, and D), accuracy is within the range of ±15–20%. Sample E, the mixture of commercial e-liquids, showed accuracy values exceeding ±20% for laboratories EP PIP, WA, and WM. In particular, the accuracy results exceeded ±20% for sample A, the lowest nicotine concentration (0.25 mg/mL), close to the LOQ (limit of quantification) value of the method (0.20 mg/mL).

Table 2Accuracy (%)* GC-MS results for nicotine, glycerol, and propylene glycol across samples and laboratories worldwide from WHO TobLabNet and EU-JATC, in May 2020

**Table:** 

*Nicotine accuracy*								
*Samples*	*Lab code*							
	*EM %*	*EP PIP %*	*EP WEI %*	*WA %*	*WC %*	*WL %*	*WM %*	*UE %*
A (A1–A2)	73	154	176	200	660	8	200	246
B (B1–B2)	88	92	94	117	102	103	103	97
C (C1–C2)	74	83	81	108	81	87	101	82
D (D1–D2)	93	111	104	117	96	108	108	113
E (E1–E2)	103	123	107	144	112	119	135	110

**Table:** 

*Glycerol accuracy*						
*Samples*	*Lab code*					
	*EP PIP %*	*EP WEI %*	*WA %*	*WC %*	*WL %*	*UE %*
A (A1–A2)	87	101	60	100	98	104
B (B1–B2)	100	103	84	107	100	87
C (C1–C2)	90	94	56	97	94	100
D (D1–D2)	99	100	74	92	99	97
E (E1–E2)	101	101	77	105	101	82

**Table:** 

*Propylene glycol accuracy*							
*Samples*	*Lab code*						
	*EP PIP %*	*EP WEI %*	*WA %*	*WC %*	*WL %*	*UD %*	*UE %*
A (A1–A2)	87	97	122	99	102	116	108
B (B1–B2)	88	92	98	104	104		108
C (C1–C2)	93	100	117	95	99		138
D (D1–D2)	88	95	101	94	100		113
E (E1–E2)	95	94	124	104	104		113

* Note that some values exceed 100%, being calculated as a difference from the ‘real’ value.

Most of the glycerol and propylene glycol accuracy results were within ±20%. Only laboratory WA results for glycerol exceeded this value. For propylene glycol, three values exceed ±20%, and two belong to the WA laboratory. Compared to the nicotine accuracy results, glycerol and propylene glycol accuracy values were generally closer to declared values. The LOQ of glycerol and propylene glycol are 2 mg/mL and 1 mg/mL, respectively.

Most precision results were under 15% for all three analytes. Table 3 reports that three samples exhibited inter-laboratory precision values exceeding 15%. Of these, two had a coefficient of variation (CV%) of 18% and 16%, which may be deemed acceptable. Again, precision for nicotine in sample A, the sample prepared below the working range of 1 mg/mL of the procedure, resulted in a high CV% of 91%, showing a loss of precision at very low analyte concentrations.

**Table 3:** Precision GC-MS results for nicotine, glycerol, and propylene glycol across samples and laboratories worldwide from WHO TobLabNet and EU-JATC, in May 2020

*Samples*	*Nicotine*			*Glycerol*			*Propylene glycol*		
	*Mean (mg/mL)*	*SD*	*CV%*	*Mean (mg/mL)*	*SD*	*CV%*	*Mean (mg/mL)*	*SD*	*CV%*
A (A1–A2)	0.54	0.49	91	520.39	93.28	18	594.25	67.46	11
B (B1–B2)	5.06	0.46	9	207.18	19.63	9	843.93	65.11	8
C (C1–C2)	8.25	1.07	13	682.34	125.64	18	297.30	48.01	16
D (D1–D2)	22.60	1.77	8	300.11	32.41	11	739.00	63.87	9
E (E1–E2)	11.91	1.42	12	339.73	42.13	12	602.52	63.83	11

CV: coefficient of variation.

The Cochran test and the Grubbs test were used to identify outliers among laboratory results for samples A-E for nicotine, glycerol, and propylene glycol (Table 4).

**Table 4:** Identification of outliers by using the Cochran test and the Grubbs test for identifying outliers among laboratories results for samples A-E for nicotine, glycerol, and propylene glycol, in May 2020

*Nicotine*					
*Lab Code*	*Sample A*	*Sample B*	*Sample C*	*Sample D*	*Sample E*
EM-MS					
EP-MS PIP					
EP-MS WEI			C**		
WA-MS					C**
WC-MS	G**/C*				
WL-MS					
WM-MS			C*		
UE-MS					
***Glycerol***					
***Lab Code***	***Sample A***	***Sample B***	***Sample C***	***Sample D***	***Sample E***
EP-MS PIP					
EP-MS WEI					
WA-MS	G*		G**	G**	
WC-MS					
WL-MS					
UE-MS					
***Propylene glycol***					
***Lab Code***	***Sample A***	***Sample B***	***Sample C***	***Sample D***	***Sample E***
EP-MS PIP					
EP-MS WEI					
WA-MS			C**		C*
WC-MS					
WL-MS					
UE-MS					

C*: Cochran straggler. C**: Cochran outlier. G*: Grubbs straggler. G**: Grubbs outlier.

### Comparison between two methods: FID versus MS

**Figure 4:**
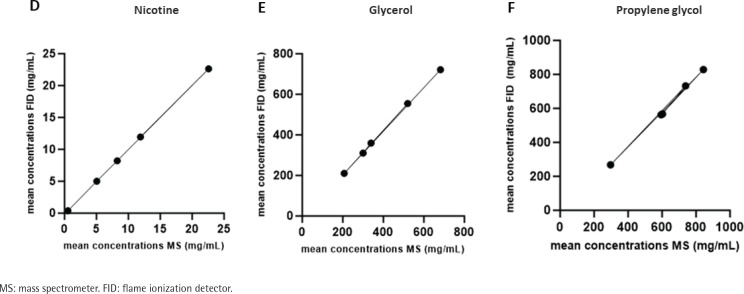
Linear correlation (Pearson’s r≃1) between mean MS and mean FID concentration (mg/mL) for nicotine (D), glycerol (E), and propylene glycol (F)

The Pearson correlation test was applied to analyze the relation between FID and MS data. FID and MS mean concentration for each sample was used to calculate r and p values. The FID means concentration results, reported in SOP11, were calculated excluding the outliers. We calculated the r and p correlation values based on the mean concentrations of all laboratories for all three analytes considering all data (Supplementary file Table 2). A linear correlation between MS and FID data is shown in Figure 4 (D-E-F). The three analytes have an r≃1, showing a positive correlation and p<0.0001 between GC-FID and GC-MS data (Supplementary file Table 3).

Student’s t-test was utilized to examine mean differences between FID and MS data within two groups. Comparing FID and MS results for nicotine, glycerol, and propylene glycol analysis across all five samples, Student’s t-test indicated no significant differences in any of the five conditions except for sample C for propylene glycol analysis, in which MS and FID methods did not show the same performance (Supplementary file Table 4).

## DISCUSSION

We demonstrated a comparable analytical capability in terms of accuracy and precision between MS and FID approaches. Statistical evaluation showed that a linear correlation between MS and FID methods exists, and these two approaches have the same performance for almost all samples. Mass spectrometry is demonstrated as a viable solution to broaden, in the future, the scope of this method, potentially encompassing the analysis of flavors extensively used in e-liquids, nicotine, impurities, and other components^25-28^. The main advantages of this MS-standardized method include its applicability in laboratories with only MS equipment and its potential utility for other analyte analysis, including flavor analysis, trace compound analysis, carcinogenic, mutagenic, or toxic for reproduction (CMR) analysis in liquids for electronic cigarettes. Also, the MS approach, as the FID method, could be applied to analyze e-liquids and collected aerosol generated from different devices using the procedure described in this study. Since this approach deals just with an extension of an original FID, there are no main limitations in using mass spectrometry compared to FID, besides the fact that the method is more complex and the staff require additional training, but laboratories that already use MS have personnel who are already trained.

This study highlights the importance of a standardized operating procedure (SOP) and collaboration among independent laboratories. This collaboration is crucial to driving the implementation of directives and ensuring the safety of tobacco-related products.

## CONCLUSIONS

Both the independent EU-JATC SOP and the WHO TobLabNet SOP11 provide comparable analytical capabilities regarding accuracy and precision for all analytes when using GC coupled with mass spectrometry, similar to traditional FID detection. This standardized method is essential for ensuring the safety and regulation of e-cigarettes and assessing the degree of agreement among independent laboratories. This is particularly important for EU-MS regulators and laboratories, as it broadens the application of the standard procedure to facilities that only have access to mass spectrometry (MS), such as university or research center laboratories, rather than flame ionization detector (FID).

## Supplementary Material



## Data Availability

The data that support the findings of this study are openly available in Zenodo (https://zenodo.org).
