# Supplementary material for: On the extension of the use of a standard operating procedure for nicotine, glycerol and propylene glycol analysis in e-liquids using mass spectrometry
Source: Tob Induc Dis. 2024 Sep 11;22:10.18332/tid/191823. doi: 10.18332/tid/191823 (PMC11389164; doi:10.18332/tid/191823)
Supplement: Supplementary file 1 [file TID-22-157-s1.pdf]

Supplementary materials

Table 1. GC-MS Laboratories list: GC column and detector's type used

Laboratory code	Type of detection	Type of GC column	Details
UE	MS	Agilent DB-ALC1 (30 m x 0.32 mm,1.8 μm)	
EM	MS	Capillary VF-35 Ms 30m x 0.25 mm x 0.25 μm	For nicotine
EP	MS	DB-1701 / Capillary Column / 60m x 0.25mm x 0.25μm	Pipetting e-liquids
			Weighting e-liquids
WM	MS	Capillary column 5% Phenyl Polysilphenylene-siloxane	For nicotine
WA	MS	1. Agilent DB-ALC1 (30 m x 0.32mm, 1.8 μm) and 2. ZB-WAX plus column (30m x 0.25mm x0.25um)	
WC	MS	Restek Rtx-BAC1, 30 m, 0.32 mm, 1.8 μm	
WL	MS	DB-WAX 30m x 0.32mm, 0.25um	
UA	MS		
UD	MS	DB-UI 8270D (30 m × 0.250 mm, 0.25 μm)	

Table 2. List of three analytes mean concentration for A-E samples

NIC (mg/mL)		
Samples	mean concentrations MS	mean concentrations FID
A	0.54	0.38
B	5.06	5.01
C	8.25	8.21
D	22.60	22.63
E	11.91	11.95
GLY (mg/mL)		
Samples	mean concentrations MS	mean concentrations FID
A	520.39	555.24
B	207.18	210.06
C	682.34	721.59
D	300.11	310.50
E	339.73	359.45
PG (mg/mL)		
Samples	mean concentrations MS	mean concentrations FID
A	594.25	562.66

B	843.93	828.96
C	297.30	268.05
D	739.00	733.31
E	602.52	567.01

Table 3. Correlation test between FID and MS results: r value and p value

Analyte	r value	p value
**Nicotine**	1	<0.0001
**Glycerol**	0.9998	<0.0001
**PG**	0.9989	<0.0001

Table 4. t Test: MS results vs FID results

**t- Test parametric version**	
**Nicotine**	
**Samples**	**P value**
A	0.31878
B	0.798285
C	0.948422
D	0.978187
E	0.969839
**t- Test parametric version**	
**Glycerol**	
**Samples**	**P value**
A	0.249624
B	0.780977
C	0.331352
D	0.426132
E	0.11095
**t- Test parametric version**	
**Glycerol**	
**Samples**	**P value**
B	0.538282
C	0.008483
D	0.713768
**t- Test non parametric version**	
**Glycerol**	
**Samples**	**P value**
A	0.257555
E	0.224

©2024 Turina A. et al.
